# Outbreak of Legionnaires’ Disease Linked to Newly Installed Residential Water Heaters, the Netherlands, 2022–2023

**DOI:** 10.3201/eid3207.260171

**Published:** 2026-07

**Authors:** Daphne F.M. Reukers, Sjoerd M. Euser, Alvin A. Bartels, Martijn G. Keet, Marijke Boot, Wilhelmina L.M. Ruijs, Petra S. Brandsema

**Affiliations:** Centre for Infectious Disease Control, National Institute for Public Health and the Environment (RIVM), Bilthoven, the Netherlands (D.F.M. Reukers, A.A. Bartels, M.G. Keet, M. Boot, W.L.M. Ruijs, P.S. Brandsema); Regional Public Health Laboratory Kennemerland, Haarlem, the Netherlands (S.M. Euser)

**Keywords:** Legionnaire’s disease, bacteria, Legionella pneumophila, respiratory infections, outbreak, water system, water heater, private homes, the Netherlands

## Abstract

During 2022–2023, two small Legionnaires’ disease (LD) clusters (2 and 4 cases) occurred in 2 residential apartment buildings in the Netherlands. All case-patients recently installed a new brand A water heater. Environmental sampling revealed *Legionella pneumophila* serogroup 1 sequence type 37 in the hot water system of each case-patient’s apartment, matching 1 clinical isolate. We conducted a case–control study to evaluate whether brand A water heaters were linked to cases in the 2 clusters. We identified 23 LD case-patients, 21 of whom had a brand A water heater installed <6 months before illness onset. Four cases had a genotypic match between clinical and environmental isolates; none of 31 control-patients had recently installed a water heater. Analyses showed that LD cases were strongly associated with new brand A water heaters (OR 542 [95% CI 24.76–11,854.03]); the manufacturer implemented control measures. Residential water heaters could serve as *L. pneumophila* transmission sources.

Legionnaires’ disease (LD) is pneumonia caused by infection with *Legionella* spp. bacteria (*1*). Legionellae can be found in water and soil in natural environments or in water installations, such as tap water, cooling towers, or wastewater treatment plants (*2*–*4*). If water from those systems or small soil particles containing *Legionella* spp. bacteria are aerosolized, the aerosols can be inhaled and subsequently cause disease. More than 60 different *Legionella* species have been identified, half of which are known to be pathogenic (*5*,*6*). In most countries, including the Netherlands, LD is primarily caused by *L. pneumophila*, of which serogroup 1 (SG1) is detected most often in patients (*7*,*8*).

LD is a notifiable disease in the Netherlands, and LD incidence has been increasing in the Netherlands and in Europe. In 2023, LD incidence reached a peak of 5.0 notifications/100,000 inhabitants in the Netherlands and 3.2 notifications/100,000 inhabitants in Europe (*8*). LD has a seasonal pattern that peaks in summer in most countries in Europe, including the Netherlands, likely because of favorable weather conditions for legionellae transmission from environmental sources (*7*,*8*). 

In the Netherlands, most LD notifications are sporadic (i.e., not cluster or outbreak-related), and the source of infection, despite source tracing, remains unknown. In the Netherlands, only 1%–2% of LD cases have a source confirmed by a genotypic match between the patient’s isolate and an environmental isolate (*7*); showers and drinking water systems in patients’ homes are the most often sampled sources for LD cases, representing 31% of sampled sources during 2013–2022 (*7*). However, on average, <1 case/year had a genotypic match to the home water system (*7*).

At the beginning of 2023, two small clusters of LD cases emerged within 2 residential apartment buildings in the Netherlands. Cluster 1 included 4 cases in 1 building, and cluster 2 had 2 cases in another building. Because >1 LD case is rarely notified from a residential building, the unusual event of 2 clusters in a short period was reason for further investigation. 

For 1 case, a clinical isolate was available and typed as *L. pneumophila* SG1 sequence type (ST) 37. ST37 is not common in drinking water in the Netherlands and is not often identified among LD patients in the country (*7*). Environmental sampling in patients’ homes revealed ST37 in the hot water systems of case-patients in both buildings, which matched with 1 clinical isolate. The concentration of *L. pneumophila* bacteria in most samples was 100–1,000 CFU/L, and it was detected in taps and shower hoses. At the building of cluster 1, the drinking water company, a certified water consultancy company, and the owners’ association of the apartment building conducted an extensive examination of the building’s hot and cold water systems. No legionellae were detected in the water samples from plumbing entering the building or the cold water samples in the apartments, except for 1 cold water sample from a mixed water tap. In addition, the building of cluster 2 was served by a different drinking water company; therefore, the drinking water supply was ruled out as the primary source. Furthermore, the drinking water company could not identify any incidents or repairs to the water supply that might have caused contamination from groundwater. 

In view of the short period (2.5 months) in which the patients fell ill and the age of the buildings (16 years), previously existing defects were unlikely to be the primary cause. The only recent change in the apartments that could explain the LD clusters was replacement of the water heaters in each apartment; all LD patients had installed a new water heater of the same brand (brand A) <3 months before onset of illness. During the examination of the water system for cluster 1, cluster 2 emerged in another building where the same brand A water heaters were installed <2 months before illness onset. Here, we describe a case–control study that we initiated to investigate whether more LD cases in the Netherlands were linked to newly installed brand A water heaters.

## Methods

In the Netherlands, all laboratory-confirmed LD cases are notifiable and reported by clinicians or medical microbiologists to the public health service (PHS). The PHS uses a structured questionnaire to conduct a source finding investigation and collects all relevant data, including demographic, diagnostic, underlying conditions, smoking, travel history, and all possible locations, activities, or other potential sources of exposure in the 14 days before illness onset. Using those data, the PHS in collaboration with the National Reference Laboratory for *Legionella* (*Legionella* Source Identification Unit) decides which water or soil samples should be collected from potential sources of infection. The reference laboratory collects and analyzes all samples, including genotyping of *Legionella* isolates.

### Case Definitions and Epidemiologic Investigation

We initiated a case–control study at the beginning of 2023. All reported LD cases included in this study were laboratory-confirmed according to the 2018 European Union/European Economic Area case definition (*9*). The PHS obtained production dates of the water heaters from the serial numbers. Because the water heaters implicated in the 2 LD clusters were all produced in 2022, we included LD patients reported from 2022 on; therefore, we retrospectively conducted some case and control finding. 

We defined cases as LD patients reported in the Netherlands during 2022–2023 who had *L. pneumophila* SG1 ST37 detected in a clinical isolate or in the water system of the patient’s home, which included the 6 cases from clusters 1 and 2. We defined controls as reported LD cases during 2021–2023 without *L. pneumophila* ST37 identified (i.e., a non-ST37 clinical isolate or no clinical isolate), for which the residential water system tested *L. pneumophila* ST37–negative or had low water temperature or a technical problem reported in the 14 days before onset of illness. We added the criteria on water heaters to obtain enough controls because we retrospectively selected controls and environmental sampling might not have been considered necessary at that time.

### Water Heater Inquiries

For cases and controls, the PHS made additional inquiries about water heaters <6 months before illness onset. Those inquiries collected information on the brand and type, installation date, setting (e.g., tap water temperature, ECO setting designed to save energy), and any issues or errors with the heater remembered by the patient or by their legal representative in case the patient was unable to provide information.

### Environmental Sampling and Microbiological Investigation

The reference laboratory collected environmental samples, then genotyped those samples and clinical isolates by using sequence-based typing and compared against European Working Group for *Legionella* Infections sequence-based typing database (*10*). To increase typing resolution, we calculated molecular serogroups, multilocus sequence typing (MLST) STs, and 1,521 locus core genome MLST (cgMLST) complex types in Seqsphere+ software version 7.7.5 (Ridom, https://www.ridom.de) by automated allele submission to the *Legionella pneumophila* cgMLST server (*11*,*12*). 

We used allelic profiles to calculate distance matrices by using a Hamming distance and ignoring pairwise missing loci. We used the allelic profile output to create minimum-spanning neighbor-joining trees on the basis of 1,535 core genomes, including the 7 housekeeping genes for sequence-based MLST typing and 1,521 genomes for cgMLST. For context, we added cgMLST results of 3 randomly selected clinical ST37 isolates (1 each from 2012, 2015, and 2019) to the minimum-spanning tree.

### Statistical Analyses

We calculated odds ratios (ORs), SEs, and 95% CIs to measure the strength of the association between newly installed brand A water heaters and LD cases with a ST37 clinical isolate, environmental sample, or both. Because >1 cell frequency was 0, we applied a Haldane-Anscombe correction (i.e., addition of 0.5 to each cell of the 2 × 2 table) (*13*,*14*). As a sensitivity analysis, we calculated an OR assuming all controls with missing data on the water heating system had a newly installed brand A water heater.

## Results

### Identification of Cases and Controls

**Figure 1:**
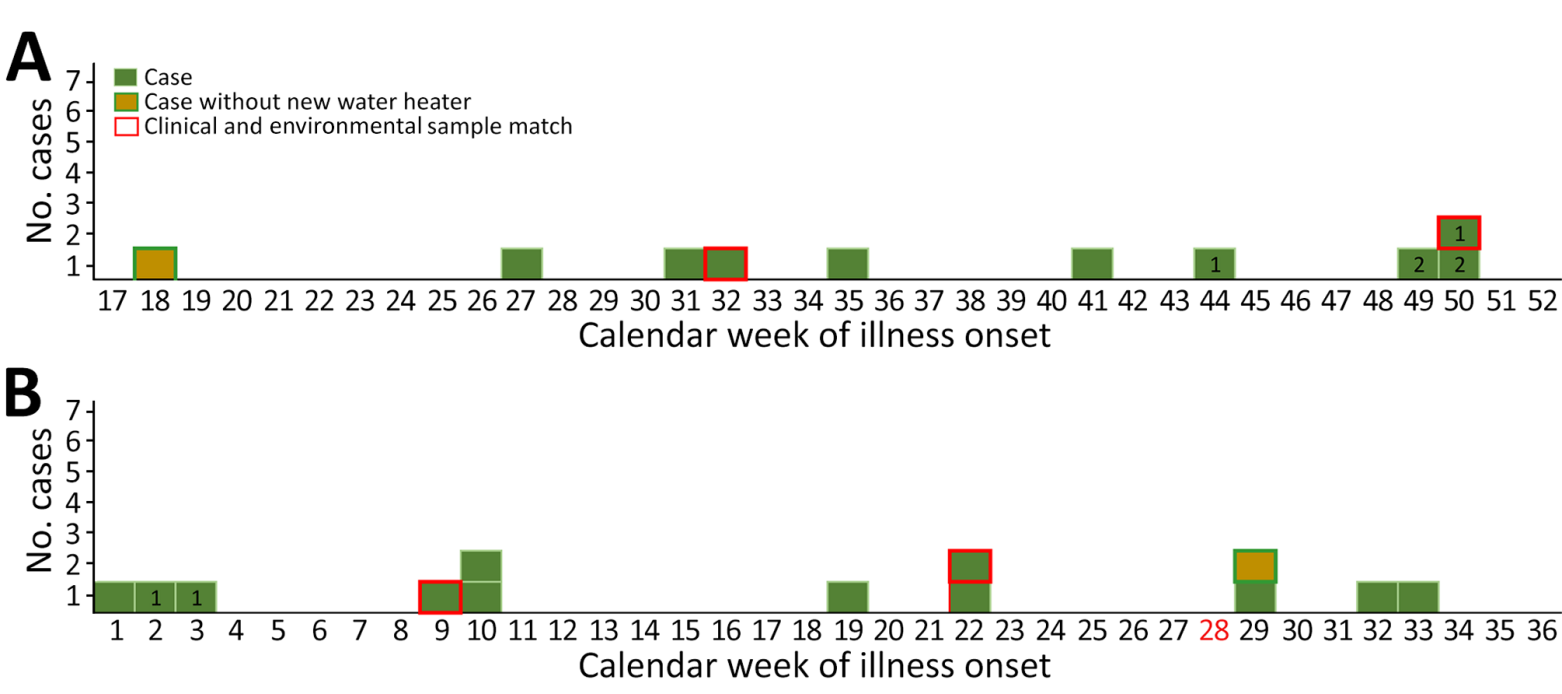
Timeline of cases in an outbreak of Legionnaires’ disease linked to newly installed residential water heaters, the Netherlands, 2022–2023. Timelines show calendar weeks for case-patient illness onset in 2022 (A) and 2023 (B). Numbered cases are from 2 clusters (cluster 1 and cluster 2) in separate apartment buildings in the Netherlands. Red date is week public warning was issued.

We identified 23 LD cases with an illness onset date during May 2022–August 2023 (Figure 1). The median age of case-patients was 76 years; 10 (43%) were male and 13 (57%) were female. All case-patients were hospitalized, and 4 (17%) died. Underlying conditions were reported for 16 (70%) and smoking for 5 (22%) case-patients. All LD cases were diagnosed by urine antigen test, 2 were also diagnosed by PCR, and 8 had an ST37 clinical isolate available (Table).

**Table:** Case characteristics in outbreak of Legionnaires’ disease linked to newly installed residential water heaters, the Netherlands, 2022–2023*

Case no.	Cluster no.	Illness onset date	Age, y/sex	Died	Diagnostic method	*Legionella pneumophila* serogroup 1 detected, sequence type			New brand A water heater	Installation date
Clinical	Environmental	Match†
1	1	2022 Nov	76/F	N	UAT	Y, ND	Y, ST37	N	Y	2022 Oct
2	1	2022 Dec	70/F	N	Culture, UAT	Y, ST37	Y, ST37	Y	Y	2022 Oct
3	1	2023 Jan	73/F	N	UAT	Y, ND	Y, ST37	N	Y	2023 Jan
4	1	2023 Jan	84/F	N	UAT	Y, ND	Y, ST37	N	Y	2022 Oct
5	2	2022 Dec	70/M	N	UAT	Y, ND	Y, ST37	N	Y	2022 Oct
6	2	2022 Dec	63/M	Y	UAT	Y, ND	Y, ST37	N	Y	2022 Oct
7	NA	2022 May	59/M	N	Culture, UAT	Y, ST37	No sampling	NA	N; probable travel-associated case	NA
8	NA	2022 Jul	88/M	N	Culture, UAT	Y, ST37	No sampling	NA	Y	2022 Jul
9	NA	2022 Aug	62/M	N	Culture, PCR, UAT	Y, ST37	No sampling	NA	Y	2022 Jul
10	NA	2022 Sep	90/M	Y	UAT	Y, ND	Y, ST37	N	Y	2022 Jun
11	NA	2022 Oct	57/M	N	UAT	Y, ND	Y, ST37	N	Y	2022 Oct
12	NA	2023 Jan	85/M	Y	UAT	Y, ND	Y, ST37	N	Y	2022 Nov
13	NA	2023 Feb	76/F	N	Culture, UAT	Y, ST37	Y, ST37	Y	Y	2022 Aug
14	NA	2023 Feb	82/F	Y	Culture, UAT	Y, ST37	Y, ST37	Y	Y	2022 Oct
15	NA	2023 Mar	93/M	N	UAT	Y, ND	Y, ST37	N	Y	2023 Jan
16	NA	2023 Mar	71/F	N	UAT	Y, ND	Y, ST37	N	Y	2023 Jan
17	NA	2023 May	55/F	N	UAT	Y, ND	Y, ST37	N	Y	2023 Mar
18	NA	2023 May	69/M	N	UAT	Y, ND	Y, ST37	N	Y	2023 Apr
19	NA	2023 Jun	77/F	N	Culture, PCR, UAT	Y, ST37	Y, ST37	Y	Y	<6 mo before illness onset
20	NA	2023 Jul	58/F	N	UAT	Y, ND	Y, ST37	N	Y	2023 Mar
21	NA	2023 Jul	81/F	N	Culture, UAT	Y, ST37	Negative	N	N	>2 y before illness onset
22	NA	2023 Aug	80/F	N	UAT	Y, ND	Y, ST37	N	Y	2023 Jul
23	NA	2023 Aug	77/F	N	UAT	Y, ND	Y, ST37	N	Y	2023 Jul

*NA, not applicable; ND, not determined; ST, sequence type; UAT, urinary antigen test. †Genotypical match between the clinical and environmental isolate, both containing *Legionella pneumophila* serogroup 1 sequence type 37.

We identified a total of 56 persons with illness onset during December 2021–May 2023. The PHS was able to collect information about the home water heaters for 31 of those patients, which we included as controls; 14 (45%) of them had reported technical problems with the water heater, and 17 (55%) had *L. pneumophila* ST37–negative home sampling. The median age of the 31 control-patients was 70 years; 18 (58%) were male and 13 (42%) were female. Most (97%, 30/31) control-patients were hospitalized, and 3 (10%) died. Underlying conditions were reported for 14 (45%) control-patients and smoking was reported for 12 (39%). Most (28/31; 90%) control cases were diagnosed using urine antigen test, 3 (10%) were diagnosed with PCR, and 7 (23%) had a clinical isolate available.

### Environmental and Microbiological Investigation

**Figure 2:**
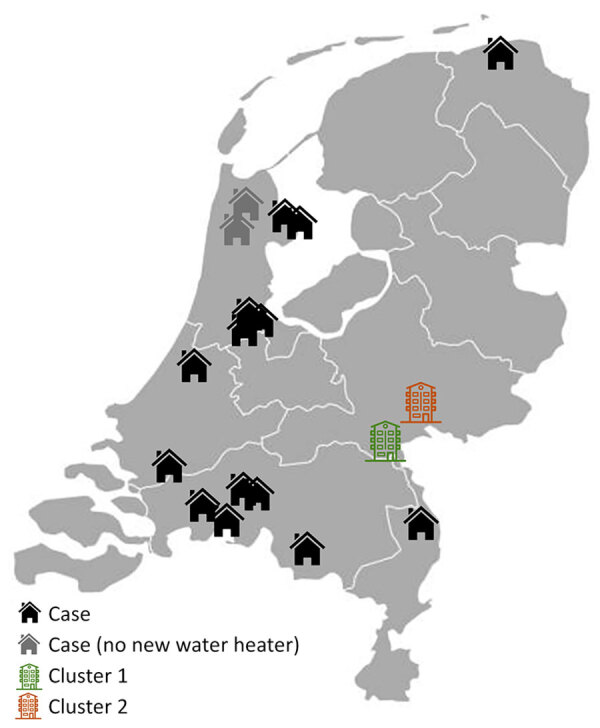
Locations of Legionnaires’ disease cases linked to newly installed residential water heaters, the Netherlands, 2022–2023. Two clusters (cluster 1 [4 cases] and cluster 2 [2 cases]) were identified in 2 apartment buildings.

Among the 23 case-patients, 21 (91%) had a brand A water heater installed <6 months before illness onset (Table, Figure 1). Two cases had a different water heater brand; 1 had traveled abroad, and their infection likely was travel-associated. Environmental sampling was conducted for homes of 21 cases, and *L. pneumophila* ST37 was detected in 20 (95%), all of which had a brand A water heater. No legionellae were detected in the home of the case-patient who had a different water heater brand. The homes of the travel-associated case-patient and 1 case-patient with a brand A water heater could not be sampled. Of the 8 cases with a clinical *L. pneumophila* ST37 isolate, 4 had a genotypic match with environmental samples from the home water system; environmental sampling of the home was not performed for 3 cases, and for 1 case, the home sample was negative. The cases were geographically spread across the Netherlands (Figure 2).

### Odds of LD

None of the 31 controls had installed a new water heater of any brand <6 months before illness onset. Analysis showed that LD cases had a statistically significant association with a newly installed brand A water heater (OR 541.80 [95% CI 24.76–11,854.03]). That association might be overestimated because we identified the 56 controls retrospectively, and 25 could not be contacted. However, we conducted a sensitivity analysis by using the assumption that all 25 controls with missing data had a newly installed brand A water heater, which still showed a statistically significant association (OR 13.02 [95% CI 2.78–60.92]).

### Whole-Genome Sequencing

**Figure 3:**
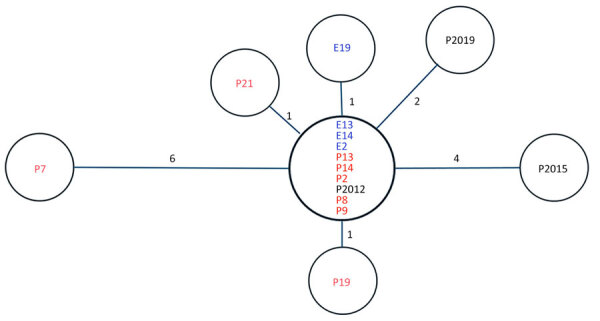
Genomic links among *Legionella pneumophila* isolates from reference sources and from an outbreak of Legionnaires’ disease linked to newly installed residential water heaters, the Netherlands, 2022–2023. Minimum-spanning tree was based on 1,521 core genome sequencing typing targets showing genomic links between *L. pneumophila* isolates from 8 clinical (red font) and 4 environmental (blue font) sequence type 37 isolates related to the outbreak, as well as 3 randomly selected non–outbreak-related sequence type 37 isolates from 2012, 2015, and 2019 for context (black font). Numbers on nodes indicate allele differences.

We included 8 *L. pneumophila* ST37 isolates from LD cases related to this outbreak and 4 environmental isolates collected during source investigations for this outbreak in the whole-genome sequencing analysis. In addition, we included 3 *L. pneumophila* ST37 isolates unrelated to this outbreak from LD cases reported in 2012, 2015, and 2019 for context. All isolates showed close relationships on the basis of cgMLST results and had a maximum of 6 alleles difference (Figure 3).

### Outbreak Control Measures

The National Institute for Public Health and the Environment informed the Netherlands Food and Consumer Product Safety Authority (NVWA) in early 2023 about the 2 LD clusters. After the case–control study and additional research at the water heater distributor in the Netherlands, the NVWA and the manufacturer issued safety warnings on July 13, 2023. The NVWA ordered the distributor to contact all clients with brand A water heaters produced during January 1, 2022–March 31, 2023, and to take necessary actions. The company implemented control measures, which included chemical disinfection of the residential water systems and thermostatic mixer valves. Pending that disinfection, clients received a shower head with a filter to prevent exposure to legionellae.

## Discussion

Newly installed brand A water heaters were identified as the most probable source of infection in this outbreak because all the available data from epidemiologic and environmental investigations showed a clear link between the water heaters and cases of *L. pneumophila* ST37 infection. Our case–control study confirmed that cases were associated with newly installed brand A water heaters with a large and highly significant OR. However, because of the partially retrospective design, data on the brand of water heater were missing for many (25/56) controls. Therefore, we performed a sensitivity analysis assuming strong bias toward the null, which still showed a statistically significant association (OR 13.02 [95% CI 2.78–60.92]). Of note, the *L. pneumophila* ST37 patient isolates and environmental strains found in this cluster were also closely related by cgMLST to isolates from 3 LD cases reported in 2012, 2015, and 2019 in patients not related to this cluster. That finding aligns with previous studies reporting on the genomic population structure of *L. pneumophila* isolates that made similar observations for some STs, indicating that isolates can be genetically closely related but not epidemiologically linked (*15*–*17*).

At the start of the outbreak, the drinking water supply was also considered as a possible source of infection but was swiftly ruled out after assessing the geographic distribution of the cases, the ORs, and detection of *L. pneumophila* ST37 only in warm, not cold, water samples. The geographic distribution of the LD cases and their homes across the country showed that they were connected to various drinking water companies and nothing indicated that *L. pneumophila* ST37 was in drinking water supplied by those companies. That observation is supported by environmental samples taken during 2012–2021 as part of source investigation by the reference laboratory of the Netherlands. The reference laboratory sampled 984 sources, including the homes of 316 LD patients, and found *L. pneumophila* ST37 in only 0.4% of all sources (*18*,*19*). Furthermore, only 3.4% of *L. pneumophila* clinical isolates during that period were ST37 (*18*,*19*). Of note, the sampling results in cluster 1 indicated this strain possibly has a high virulence because a low concentration (<1.000 CFU/L) of ST37 caused multiple LD cases. Therefore, ST37 would have been found more often in potential sources or in the clinical isolates if the strain were commonly in drinking water in the Netherlands, even in low concentrations. Finally, we also looked at possible introduction of ST37 in the drinking water system by other sources. In the building of cluster 1, a device to control the water pressure had been replaced during 2022, but that device was not replaced in the building in cluster 2 or for any of the other cases. Source finding history did not show any other sources of *L. pneumophila* ST37 introduction into the water system of the homes of the patients or any other common sources of exposure.

On the basis of the questionnaire and temperatures measured at sampling, we did not find specific risk factors related to the temperature settings or other common issues or errors with the water heaters. At most homes, the hot water temperatures at the tap were >55°C. Some water heaters were set on Eco mode (energy efficient), resulting in low temperatures for the first few minutes. How often and for how long the patients used the hot water is not known. 

The brand A water heater is also sold in several other countries in Europe. To identify possible cases in those countries, we issued a warning through EpiPulse, an online infectious disease surveillance portal for Europe managed by the ECOC, but no other countries reported LD cases. However, several countries communicated that they might be unable to detect such cases because detailed case finding information with environmental investigations and typing of isolates are not routinely performed in those countries. Furthermore, unlike the Netherlands, many countries use biocides in their drinking water system, which could explain why no cases have been observed in other countries. In the Netherlands, chemical or thermal disinfection is also not mandatory after water heater installation, which could explain why no cases have been observed in other countries.

The brand A water heater had a flow-through system without a storage tank, so the legionellae growth cannot be explained by stagnant water with temperatures of 25°C–50°C degrees or thermal stratification in the hot water storage tank, which is known to pose a higher risk for legionellae growth (*20*). However, a plausible explanation for introduction of legionellae into the water system is that a small amount of test water containing *L. pneumophila* ST37 remained in the heater after the production process and that microorganisms were able to create a biofilm, enabling *L. pneumophila* to survive. All heaters are tested with water for possible leakage before distribution. During the investigation into the clusters, the reference laboratory confirmed that a small (≈30 mL) amount of water was in new water heaters still in storage. We hypothesize that after water heater installation, *L. pneumophila* was released into the hot water system in the home and was able to grow in the pipes, shower hoses, and shower heads, subsequently causing infection. However, before the safety warning, no *L. pneumophila* ST37 was detected by the reference laboratory in samples from the small amount of water in the water heaters still in storage.

Further research is necessary to assess the risk for *L. pneumophila* contamination in water heaters causing infections and provide evidence-based policy recommendations for infection prevention and control. Previous research has documented that *L. pneumophila* is able to enter a viable but nonculturable state and later resuscitate in the right environment (*21*,*22*), which might explain how the bacteria in the LD clusters survived in storage for months. However, more data are needed to elucidate how *L. pneumophila* ST37 entered the water heaters, how the bacteria were able to grow in the plumbing system despite high water temperatures, and whether energy-saving designs like Eco Mode result in lower temperatures and an increased risk for bacterial growth. Further research also is needed to determine whether *L. pneumophila* infection is specific to brand A water heaters or if similar infections could occur in water heaters from other brands as well.

In conclusion, *L. pneumophila* in plumbing systems is widely reported, often in relation to water temperatures of 25°C–50°C, stagnant water, or low biocide levels. The risk for legionellae growth in thermostatic valves and biofilms in shower hoses also is well known (*23*–*26*). This study demonstrated the value of legionellae control measures during production or installation of water devices and that a new device could pose a risk for introduction of *L. pneumophila* into a water system. Manufacturers of water heaters or fittings for plumbing systems that are tested with water should ensure devices are clean and dry before packaging and should control for *L. pneumophila* growth in the test water. Furthermore, this outbreak emphasizes how vital an adaptable surveillance system, environmental investigations, typing of isolates, and alert public health workers are for detecting previously unrecognized sources of *L. pneumophila*.

## References

[1] Steinert M, Hentschel U, Hacker J. *Legionella pneumophila*: an aquatic microbe goes astray. FEMS Microbiol Rev. 2002;26:149–62. 10.1111/j.1574-6976.2002.tb00607.x 12069880

[2] National Academies of Sciences, Engineering, and Medicine; Health and Medicine Division; Division on Earth and Life Studies; Board on Population Health and Public Health Practice; Board on Life Sciences; Water Science and Technology Board; Committee on Management of Legionella in Water Systems. Management of *Legionella* in water systems. Washington: National Academies Press; 2019.

[3] van Heijnsbergen E, Schalk JAC, Euser SM, Brandsema PS, den Boer JW, de Roda Husman AM. Confirmed and potential sources of *Legionella* reviewed. Environ Sci Technol. 2015;49:4797–815. 10.1021/acs.est.5b00142 25774976

[4] Orkis LT, Harrison LH, Mertz KJ, Brooks MM, Bibby KJ, Stout JE. Environmental sources of community-acquired Legionnaires’ disease: a review. Int J Hyg Environ Health. 2018;221:764–74. 10.1016/j.ijheh.2018.04.013 29729999

[5] Yu VL, Plouffe JF, Pastoris MC, Stout JE, Schousboe M, Widmer A, et al. Distribution of *Legionella* species and serogroups isolated by culture in patients with sporadic community-acquired legionellosis: an international collaborative survey. J Infect Dis. 2002;186:127–8. 10.1086/341087 12089674

[6] Fields BS, Benson RF, Besser RE. *Legionella* and Legionnaires’ disease: 25 years of investigation. Clin Microbiol Rev. 2002;15:506–26. 10.1128/CMR.15.3.506-526.2002 12097254 PMC118082

[7] Reukers DFM, Bartels AA, Mulder AC, Berry DSF, Euser S, Laarman C, et al. Surveillance of legionellosis in the Netherlands. Overview of clusters, sources and environmental factors between 2013 and 2022 [in Dutch]. Bilthoven: National Institute for Public Health and the Environment; 2024.

[8] European Centre for Disease Prevention and Control. Legionnaires’ disease. Annual epidemiological report for 2023. Stockholm: The Centre; 2026.

[9] European Commission. Case definitions of communicable diseases: Legionnaires’ disease. Official Journal of the European Union. 2018 Jul 7 [cited 2025 Jan 27]. https://eur-lex.europa.eu/legal-content/EN/TXT/PDF/?uri=CELEX:32018D0945&from=EN#page=26

[10] Gaia V, Fry NK, Afshar B, Lück PC, Meugnier H, Etienne J, et al. Consensus sequence-based scheme for epidemiological typing of clinical and environmental isolates of *Legionella pneumophila.* J Clin Microbiol. 2005;43:2047–52. 10.1128/JCM.43.5.2047-2052.2005 15872220 PMC1153775

[11] Jünemann S, Sedlazeck FJ, Prior K, Albersmeier A, John U, Kalinowski J, et al. Updating benchtop sequencing performance comparison. Nat Biotechnol. 2013;31:294–6. 10.1038/nbt.2522 23563421

[12] Moran-Gilad J, Prior K, Yakunin E, Harrison TG, Underwood A, Lazarovitch T, et al. Design and application of a core genome multilocus sequence typing scheme for investigation of Legionnaires’ disease incidents. Euro Surveill. 2015;20:28. 10.2807/1560-7917.ES2015.20.28.21186 26212142

[13] Haldane JB. The estimation and significance of the logarithm of a ratio of frequencies. Ann Hum Genet. 1956;20:309–11. 10.1111/j.1469-1809.1955.tb01285.x 13314400

[14] Anscombe FJ. On estimating binomial response relations. Biometrika. 1956;43:461–4. 10.1093/biomet/43.3-4.461

[15] Quero S, Párraga-Niño N, Barrabeig I, Sala MR, Pedro-Botet ML, Monsó E, et al. Population structure of environmental and clinical *Legionella pneumophila* isolates in Catalonia. Sci Rep. 2018;8:6241. 10.1038/s41598-018-24708-1 29674708 PMC5908911

[16] David S, Mentasti M, Tewolde R, Aslett M, Harris SR, Afshar B, et al. Evaluation of an optimal epidemiological typing scheme for *Legionella pneumophila* with whole-genome sequence data using validation guidelines. J Clin Microbiol. 2016;54:2135–48. 10.1128/JCM.00432-16 27280420 PMC4963484

[17] Schjørring S, Stegger M, Kjelsø C, Lilje B, Bangsborg JM, Petersen RF, et al.; ESCMID Study Group for Legionella Infections (ESGLI). Genomic investigation of a suspected outbreak of *Legionella pneumophila* ST82 reveals undetected heterogeneity by the present gold-standard methods, Denmark, July to November 2014. Euro Surveill. 2017;22:25. 10.2807/1560-7917.ES.2017.22.25.30558 28662761 PMC5490456

[18] Den Boer JW, Euser SM, Brandsema P, Reijnen L, Bruin JP. Results from the National *Legionella* Outbreak Detection Program, the Netherlands, 2002–2012. Emerg Infect Dis. 2015;21:1167–73. 10.3201/eid2107.141130 26079594 PMC4480379

[19] Euser SM, Bruin JP, Brandsema P, Reijnen L, Boers SA, Den Boer JW. Legionella prevention in the Netherlands: an evaluation using genotype distribution. Eur J Clin Microbiol Infect Dis. 2013;32:1017–22. 10.1007/s10096-013-1841-9 23430195

[20] Roman FA Jr, Martin RL, Rhoads WJ, Pearce A, Smeltz RE, Pruden A, et al. Water heater type, temperature setting, operational conditions, and insulation affect ecological niches for *Legionella* growth. ACS ES T Water. 2024;5:377–86. 10.1021/acsestwater.4c00894 39816977 PMC11731276

[21] Steinert M, Emödy L, Amann R, Hacker J. Resuscitation of viable but nonculturable *Legionella pneumophila* Philadelphia JR32 by *Acanthamoeba castellanii.* Appl Environ Microbiol. 1997;63:2047–53. 10.1128/aem.63.5.2047-2053.1997 9143134 PMC168494

[22] García MT, Jones S, Pelaz C, Millar RD, Abu Kwaik Y. *Acanthamoeba polyphaga* resuscitates viable non-culturable *Legionella pneumophila* after disinfection. Environ Microbiol. 2007;9:1267–77. 10.1111/j.1462-2920.2007.01245.x 17472639

[23] Whiley H, Giglio S, Bentham R. Opportunistic pathogens *Mycobacterium avium* complex (MAC) and *Legionella* spp. colonise model shower. Pathogens. 2015;4:590–8. 10.3390/pathogens4030590 26213977 PMC4584274

[24] Proctor CR, Reimann M, Vriens B, Hammes F. Biofilms in shower hoses. Water Res. 2018;131:274–86. 10.1016/j.watres.2017.12.027 29304381

[25] Hayes-Phillips D, Bentham R, Ross K, Whiley H. Factors influencing *Legionella* contamination of domestic household showers. Pathogens. 2019;8:27. 10.3390/pathogens8010027 30813532 PMC6470800

[26] Cavallaro A, Rhoads WJ, Sylvestre É, Marti T, Walser JC, Hammes F. *Legionella* relative abundance in shower hose biofilms is associated with specific microbiome members. FEMS Microbes. 2023;4:xtad016. 10.1093/femsmc/xtad016 37705999 PMC10496943

